# Differential gene expression profile reveals deregulation of pregnancy specific β1 glycoprotein 9 early during colorectal carcinogenesis

**DOI:** 10.1186/1471-2407-5-66

**Published:** 2005-06-27

**Authors:** Sima Salahshor, Jason Goncalves, Runjan Chetty, Steven Gallinger, James R Woodgett

**Affiliations:** 1 Ontario Cancer Institute, Department of Medical Biophysics, 610 University Ave., Toronto, Ontario, M5G 2M9, Canada; 2 Princess Margaret Hospital, Department of Laboratory Medicine and Pathobiology, University of Toronto, Canada; 3 Mount Sinai Hospital, Department of Surgery, Centre for Cancer Genetics, Samuel Lunenfeld Research Institute, Toronto, Canada

## Abstract

**Background:**

*APC *(Adenomatous polyposis coli) plays an important role in the pathogenesis of both familial and sporadic colorectal cancer. Patients carrying germline *APC *mutations develop multiple colonic adenomas at younger age and higher frequency than non-carrier cases which indicates that silencing of one APC allele may be sufficient to initiate the transformation process.

**Methods:**

To elucidate the biological dysregulation underlying adenoma formation we examined global gene expression profiles of adenomas and corresponding normal mucosa from an FAP patient. Differential expression of the most significant gene identified in this study was further validated by mRNA *in situ hybridization*, *reverse transcriptase *PCR and Northern blotting in different sets of adenomas, tumours and cancer cell lines.

**Results:**

Eighty four genes were differentially expressed between all adenomas and corresponding normal mucosa, while only seven genes showed differential expression within the adenomas. The first group included pregnancy specific β-1 glycoprotein 9 (PSG9) (p < 0.006). *PSG9 *is a member of the carcinoembryonic antigen (CEA)/PSG family and is produced at high levels during pregnancy, mainly by syncytiotrophoblasts. Further analysis of sporadic and familial colorectal cancer confirmed that *PSG9 *is ectopically upregulated *in vivo *by cancer cells. In total, deregulation of PSG9 mRNA was detected in 78% (14/18) of FAP adenomas and 75% (45/60) of sporadic colorectal cancer cases tested.

**Conclusion:**

Detection of *PSG9 *expression in adenomas, and at higher levels in FAP cases, indicates that germline APC mutations and defects in Wnt signalling modulate *PSG9 *expression. Since *PSG9 *is not found in the non-pregnant adult except in association with cancer, and it appears to be an early molecular event associated with colorectal cancer monitoring of its expression may be useful as a biomarker for the early detection of this disease.

## Background

FAP is characterized by the development of hundreds to thousands of adenomas throughout the entire colon and rectum which, if left untreated, progress to colorectal cancer [1,2]. FAP, an inherited tumour predisposition, is caused by mutant alleles of the adenomatous polyposis coli (APC) gene and provides an opportunity to define critical early genetic events in the development of tumours [3]. Early development of a large number of colon adenomas in this disorder indicates that mutations in the APC gene can be rate-limiting in adenoma development. The majority of colorectal tumours are sporadic in origin, however, they exhibit close similarities to tumours resulting in inherited colorectal cancer syndromes. Most sporadic colon adenomas and carcinomas also harbour APC gene mutations [4]. The APC gene, which has been recognized as a gatekeeper of colorectal carcinogenesis, is one of the key components of the Wnt signalling pathway. Wnt signalling induces nuclear translocation of transcriptionally active β-catenin through interference with the β-catenin-destruction complex, composed of glycogen synthase kinase-3 (GSK-3α and β), Axin (Axin1 and 2) and APC. In the absence of a Wnt signal this complex efficiently earmarks cytoplasmic β-catenin for degradation through the ubiquitin/proteasome pathway [5,6].

To identify the possible differences between different adenomas that either predispose to cancer or result in benign growths, we compared variations in gene expression between different adenomas and normal mucosa from the same patient with a germline mutation in the APC gene. The approach was designed to identify very early changes that occur during adenoma formation and to detect aberrant regulation of genes required for adenoma-carcinoma progression. Microarray-based expression profiling revealed that gene expression patterns between different adenomas are very similar but are different from normal mucosa. We describe the increased expression of a specific member of the pregnancy specific glycoprotein family and show that induction of this gene is a very early event that does not appear to be dependent on activation of β-catenin.

## Methods

### Samples

Adenomatous polyps, tumours and matched adjacent normal mucosal tissue samples from 18 FAP cases (germline APC mutations detected by standard techniques), 60 sporadic colorectal cancer cases, five liver metastases and one normal placenta, were obtained from University Health Network (UHN) human tissue bank and the Familial GI Cancer Registry at Mount Sinai hospital, in compliance with each Institutional Review Board. Colorectal cancer cell lines; SW620, SW480, LoVo, RKO, SW1417, LS1034 and MCF12A were purchased from ATCC and grown in media recommended by the distributor. Total RNA samples from normal ovarian, prostate, colon, breast and placental tissues were purchased from Ambion and Clontech. RNA was extracted from cell lines and tissue samples using an RNAeasy kit (Qiagen). Tissues were processed for RNA extraction, *in situ *hybridization or immunohistochemistry analysis.

### Microarray procedure and data analysis

cDNA microarrays consisting of 19,200 human gene clones were employed to explore the variation in gene expression between adenoma and normal mucosa. Microarray slides were obtained from the University Health Network Microarray Centre (UHN, Toronto, Canada). Protocols used for array hybridisation were as published on the UHN Microarray Centre web page  with some modifications. Briefly, 5 μg total RNA extracted from normal mucosa or adenoma was labelled with Cy5 and, a reference total RNA pool was labelled with Cy3. The reference RNA used was composed of total RNA from 10 human cell lines (Stratagene) which hybridize to the maximum number of spots on the array. The signals obtained from reference RNA have been used for normalisation of experimental samples. Microarray hybridization was carried out in a hybridization chamber humidified with 2XSSC. Labeled cDNA was dissolved in 80 μl of the hybridization buffer, denatured at 95°C for three minutes in a thermal cycler, and applied on the microarray slides. Microarrays were incubated overnight at 37°C. Post-hybridization washing was performed by serial incubations in buffers with decreasing SSC and SDS concentrations, at 50°C. At least two replicates including dye switches were performed for each experiment to account for possible dye labelling and hybridization bias. Relative expression was assessed by a two-colour hybridization experiment. Slides were scanned using either an Axon GenePix 4000A (Axon) or ScanArray 4000 Scanner (Packard BioScience). The scanned 16-bit TIFF images were quantified using QuantArray software (Packard BioScience). The quantified data files were transferred to a GeneTraffic microarray database and analysis system (Iobion Informatics, Stratagene) with a complete annotation of experiments based on the current MIAME standards for microarray experiments . Each hybridization dataset was filtered and spots that did not pass the quality criteria in both channels were excluded from further analysis. The lowess subarray normalization which uses a local weighted smoother to generate an intensity-dependent normalisation function was applied to each hybridization. The normalised log_2 _ratios were used for statistical analysis. Data were analysed by both SAM software (Significance Analysis for Microarrays) and the statistical program integrated into GeneTraffic 2.8. Genes exhibiting a consistent 2-fold or more up- or down regulation with a p value of <0.05 were considered significant.

### Semi-quantitative and quantitative RT-PCR

**Figure 1:**
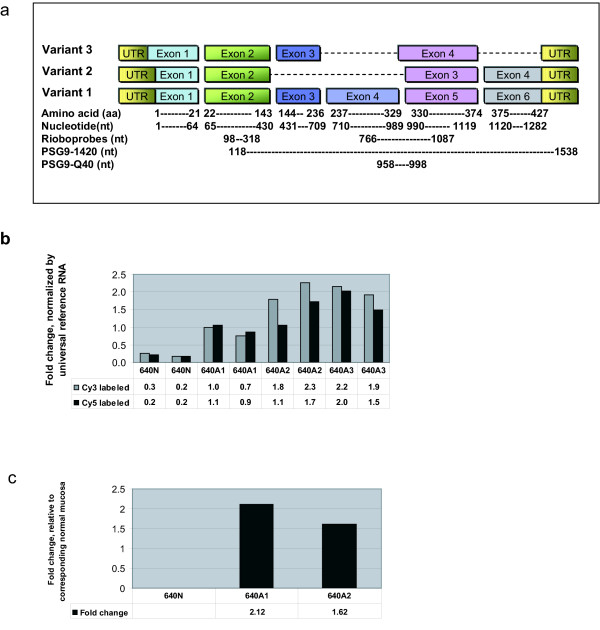
*Genomic structure of PSG9*. The human *PSG9 *gene is located on chromosome 19q13.2 and encodes at least three variants. The exon structures of each isoform are indicated in the figure. The largest PSG9 variant, variant 1, consists of 1282 nucleotides and encodes for a protein of 427 amino acids. The location and size of each primer and riboprobes used in this study are indicated (a). Statistical analysis of microarray data using SAM program revealed up to two-fold over-expression of *PSG9 *in adenoma 1, 2 and 3 (A1 to A3) compared to normal (N). The log^2 ^ratio of fluorescence intensity is shown on the y-axis while *PSG9 *expression in different samples is shown on the x-axis. At least four microarray replicates including dye switches (Cy3- or Cy5-labelled) were performed to account for possible labeling and hybridization bias (b). The data have further been verified in adenoma 1, 2 and normal tissue by *Real Time *PCR (c).

Two μg total RNA was reverse transcribed by SuperScript II reverse transcriptase (Stratagene). One μg of each cDNA were amplified using *PSG9 *specific primers (PSG9-1420) by AccuaPrime *Taq *DNA Polymerase system (Invitrogen) (Fig. 1). For semi-quantitative PCR, all samples were normalised to the level of glyceraldehyde-3-phosphate dehydrogenase (GAPDH) before PSG9 specific amplification. For quantitative real-time PCR analyses, 500 ng cDNA was amplified using a SYBR Green PCR kit and either primer *PSG9*, *GAPDH *or *Axin2 *and a 7900 Sequence Detector system (Applied Biosystems). Primers were designed by either Oligo 6.4 (Oligo) or Primer Express software (Applied Biosystems). Location of all primers for *PSG9 *are indicated in figure 1a. Primer sequences used for semi-quantitative PCR were as follow: PSG9-1420F (5'-CCA GCC ACC CAA AGT TTC-3', PSG9-1420R (5'-GGG CAT TCA GAT AGA CAG CAA-3'), GAPDH-F (5'-GAC GCC TGC TTC ACC ACC TTC-3') and GAPDH-R (5'-CCG CTT CGC TCT CTG CTC C-3'). Primers used for quantitative RT-PCR were as follows: PSG9-Q40F (5'-TGG TGG CCT CCG CAG TAA-3'), PSG9-Q40R (5'-GTC TGG ACC ATA GAG GAC ATT TAG G-3'), GAPDH-F 5'-GAA GGT GAA GGT CGG AGT C-3' and GAPDH-R 5'-GAA GAT GGT GAT GGG ATT TC-3', Axin2F (5'-CCA CAC CCT TCT CCA ATC CA-3') and Axin2R (5'-TGG ACA CCT GCC AGT TTC TTT-3'). PCR conditions are available upon request.

### Analysis of PSG9 sequence variants in tumors

PSG9 isoforms expressed in normal placenta, SW620 and LoVo colon cancer cell lines and two sporadic colorectal cancer cases, were PCR amplified. Different isoforms were separated on a 2% agarose gel and purified by PerfectPrep gel cleanup kit (Eppendorf). The purified DNA products were cloned into pCR^®^II-TOPO vector. Clones were sequenced in both directions using T7 and T3 primers using an ABI sequencing system (Applied Biosystems). Sequence variation was examined using GeneJockey II software (Biosoft).

### Northern blot analysis

*PSG9 *expression patterns were analysed using random primed radiolabeled full-length *PSG9 *cDNA. A 2 kb fragment was digested from a cDNA clone (GeneBank Accession No. 196828) by PAC I and EcoRI restriction enzymes, gel purified and then radiolabeled with [α-^32^P]dCTP using a T7 Quik Prime system (Pharmacia Biotech). Northern blot analysis was performed based on standard techniques [7].

### RNA in situ hybridisation

RNA *in situ *hybridization (RISH) was used for the examination of *PSG9 *and *PSG2 *mRNA expression. Nonradioactive RISH was applied to frozen or paraffin-embedded sections using digoxigenin (DIG)-labelled copy RNA (cRNA) probes. Different cDNA fragments corresponding to human *PSG9 *(GenBank 196828) were amplified by PCR using sense and antisense primers containing either T7, T3 or SP6 promoter sequences, respectively. *PSG2 *riboprobes were amplified from a human placental RNA pool (Clontech). The locations of primers and oligoprobes sequences are indicated in figure 1. For cRNA probe synthesis, purified PCR products were used. The transcripts were labelled with DIG-labelled nucleotides, DIG RNA labelling kit and either T7, T3 or SP6 RNA polymerase (Roche Applied Science) to produce DIG-labelled riboprobes. Primers used for amplification and synthesis of each cRNA probe were: PSG9-E2; Sense 5'-T GCC GAA GTC ACG ATT GAA G-3', Anti-sense: 5'-GGA TGC GTT GGA ATA TAC TGT TTC T-3', PSG9-E4-5; Sense: 5'-A TGT CTT AGC CTT CAC CTG TG-3', Anti-sense: 5'-AGT GCC GGT GGG TTA GAT T-3' PSG2; Sense: 5'-GTC CAG ACC TCC CCA GAA T-3', Anti-sense 5'-AGG CTG CTA TGT TGG ATT AAG GAG AG-3'. PCR conditions are available upon request. Seven-μm-thick sections of paraffin-embedded or fresh frozen tissue were cut, fixed in 1XPBS (phosphate-buffered saline) containing 4% paraformaldehyde. A standard *in situ *hybridization technique was used with some modifications. Images were analysed by light microscope (Leica).

### Western blot and immunohistochemical analysis

To detect β-catenin and β-actin, cells were lysed in RIPA buffer (150 mM NaCl, 1% NP-40, 0.5% DOC, 0.1% SDS, 50 mM Tris pH 8.0, 1 mM EDTA). Twenty μg protein were loaded onto 8% SDS polyacrylamide gels, separated and transferred to a nitrocellulose filter by semidry transfer. Western blotting were performed using a standard protocol. The antibody dilutions were as follows; primary antibodies β-catenin (1:1000; Transduction Laboratories) and β-actin (1:2000; Abcam), secondary monoclonal antibody (1:5000; BioRad). Standard immunohistochemistry (IHC) was carried out on formalin-fixed, paraffin-embedded or fresh frozen sections.

### Wnt signalling stimulation and reporter gene assay

RKO cells were treated over night with 50 nM Wnt3a recombinant (R&D systems) or 10 μM Kenpaullone (Calbiochem) an inhibitor of GSK3 [8] prior to RNA or protein extraction. RKO cells transfection was performed in six-well plates at the density of 1.5 × 10^5 ^cells/well with Lipofectamine 2000 reagent (Invitrogen). Cells were transfected with either Super8XTOP- or 8XFOPFlash and β-galactosidase constructs [9] and were assayed for luciferase activity 23 hrs post-transfection/treatment. The luciferase activity was measured and quantified in a luminometer using a chemiluminescent reporter gene assay system for the combined detection of luciferase and β-galactosidase as recommended by manufacturer (Applied Biosystems). The β-galactosidase was used to normalize luciferase units in each transfection.

## Results

### Gene expression profiling

Gene profiles of three adenomas and corresponding normal epithelial tissue from an FAP patient (case 640) with an *APC *germline mutation (n. 2092; T→G) were analysed using 19K human cDNA microarray chips. Statistical analysis using SAM and GeneTraffic 2.8 revealed eighty four transcripts to be represented at statistically significant different levels in all adenomas compared to normal (p < 0.05) (Table 1, 2, 3). *Pregnancy specific β1 glycoprotein 9 *(PSG9) showed a consistent two fold up-regulation in adenomas compared to normal mucosa and as the most statistically significant candidate in these experiments (p < 0.006) was selected for further analysis (Fig. 1b). The result was consistent between all adenomas on all the microarray chips tested. Differential *PSG9 *expression was further verified using quantitative RT-PCR (Fig. 1c). PSG9 is a member of the pregnancy specific glycoprotein family (PSGs) [10,11]. We also detected deregulation of a number of *TGFβ *(transforming growth factor β) regulated genes in our cDNA microarray gene expression screening. Similar to PSG9, TGFβ plays an essential role in normal placentation and is expressed by uterine epithelium from early in pregnancy (Table 1 and 2).

### PSG family member expression

Seventeen PSG clones are represented on the 19K human chip used in these studies: four clones represent PSG1, one for PSG3, two PSG4, two PSG5 clones, two PSG6, three PSG11, and three clones are assigned as *PSG9*. To examine why only one of the PSG9 clones on the cDNA chip indicated differential expression between normal mucosa and polyps, we re-sequenced all the clones representing PSG9. The sequencing results showed that only clone 196828 contained a full-length PSG9 cDNA. This clone represents the largest transcript of *PSG9*. Because of the high homology (>90%) between different *PSG *genes and possible cross-hybridization and non-specific binding of primers to different PSGs and also to different transcripts of *PSG9*, all primers were designed based on the sequence of this clone (Fig. 1a).

### Expression of PSG9 in normal and cancer cells

**Figure 2:**
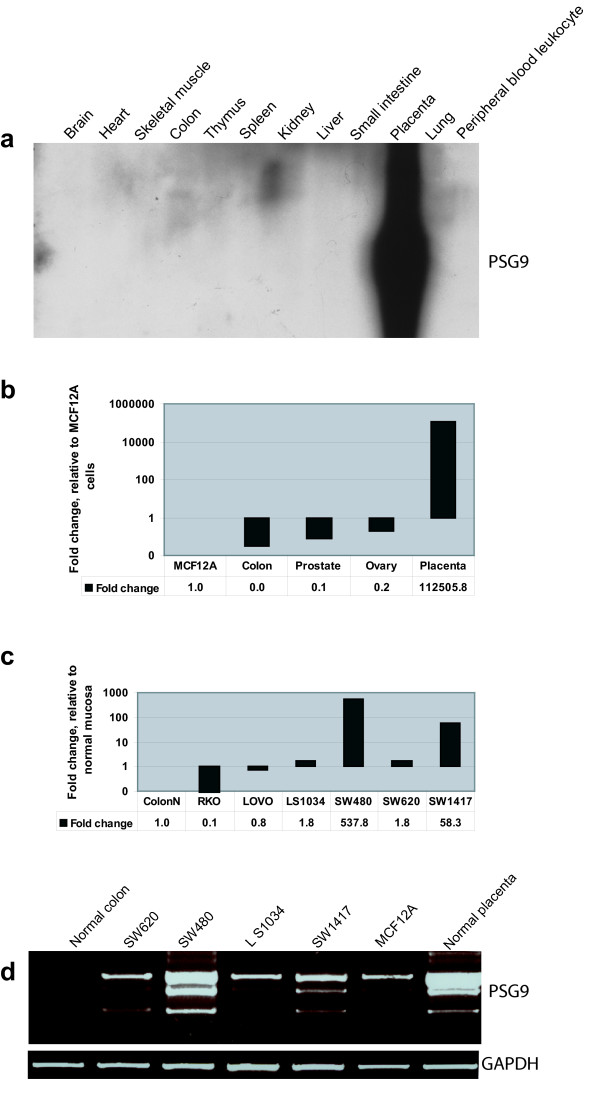
*PSG9 is exclusively expressed by placental cells*. Quantitative RT-PCR analysis of *PSG9 *transcript (a) and northern blot analysis of different normal tissues (1 μg mRNA/lane) probed with the labelled PSG9 cDNA (b) shows specific expression of *PSG9 *in placenta. (a-b). Colorectal cancer cell lines were also examined for *PSG9 *expression level by quantitative- (c) and semi-quantitative RT-PCR (d). Highest expression level of *PSG9 *was detected in SW480 cells and was lowest in the RKO cell line (c-d).

**Figure 3:**
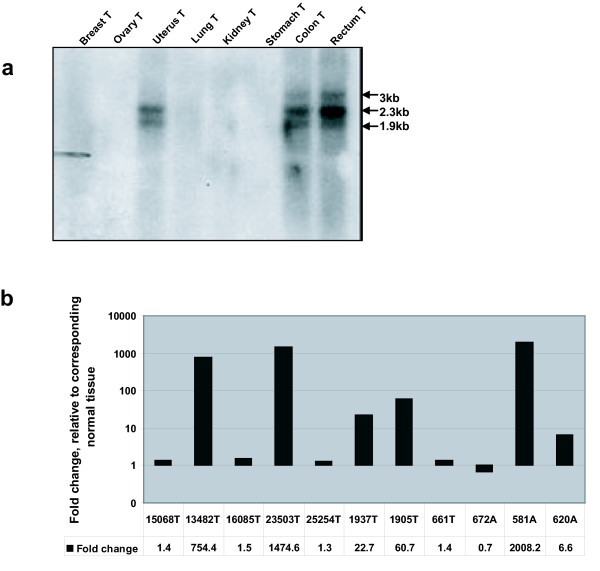
*PSG9 is ectopically expressed by cancer cells*. A multiple-tumour Northern blot (10 μg RNA/lane) revealed over-expression of *PSG9 *in colon, rectal and uterus cancer. At least three different transcripts were observed (a). Expression of PSG9 "isoform a" was examined in a panel of colorectal cancer cases. Forty-nine percent (15/27) of cases showed up-regulation of PSG9 (isoform a) in the tumours compared to corresponding normal tissue (b). However, the expression levels were different between different cases (T; tumour, A; adenoma).

PSG isoforms were originally identified in the circulation of pregnant women [12] and PSG concentrations in the bloodstream increase exponentially until term. By the third trimester the concentration reaches 200–400 μg/ml [13]. Using monoclonal antibodies against PSG as a group and studying their mRNA expression level, it was shown that *PSGs *are expressed mainly by synthiotrophoblasts during pregnancy, while their expression in normal colon, could not be detected [14,15]. Other studies have reported that PSGs are not exclusively expressed in human placenta and a number of PSG cDNA clones have been isolated from fetal liver, salivary gland, testis, myeloid cells and intestine [16-18]. However, the expression level in these tissues appears to be very low. Because anti-PSG antibodies used in some studies can cross-react with other members of the CEA family, the expression pattern and level of individual PSG proteins and their variants is not well documented. Therefore, we examined the normal expression pattern of *PSG9 *in different tissues by using a multiple tissue northern blot, semi- and quantitative RT-PCR. High expression of *PSG9 *transcripts was detected in placenta after only few hours of exposure, while expression in other tissues, including normal colon mucosa was undetectable even after one week exposures (Fig 2a). This result was further verified by quantitative RT-PCR analysis of *PSG9 *transcripts in different normal tissues (Fig. 2b). PSG9 expression was also examined in a panel of colorectal cancer cell lines. The highest level of *PSG9 *was found in the SW480 cell line which, similar to placental cells, expresses at least three different *PSG9 *transcripts. The RKO colon cancer cell line exhibited the lowest level of PSG9 (Fig. 2c–d). We also examined the degree of *PSG9 *expression in a panel of RNAs extracted from different tumours. Northern blot analysis showed clear expression of three PSG9 transcripts in colon and rectal cancer, and two transcripts in uterine cancer (Fig. 3a). Primary sporadic colorectal tumours, liver metastasis and corresponding normal tissues were also examined for *PSG9 *expression by semi- and quantitative PCR. Deregulation of PSG9 variants could be detected in most tumours and liver metastases tested. Of note, the expression pattern varied between different tumours and also between different colon cancer cell lines, which may relate to the stage of differentiation or type of mutation in those cases (Fig. 3b). It is also possible that the type of *APC *mutation plays a role in the level and pattern of *PSG9 *transcription.

**Figure 4:**
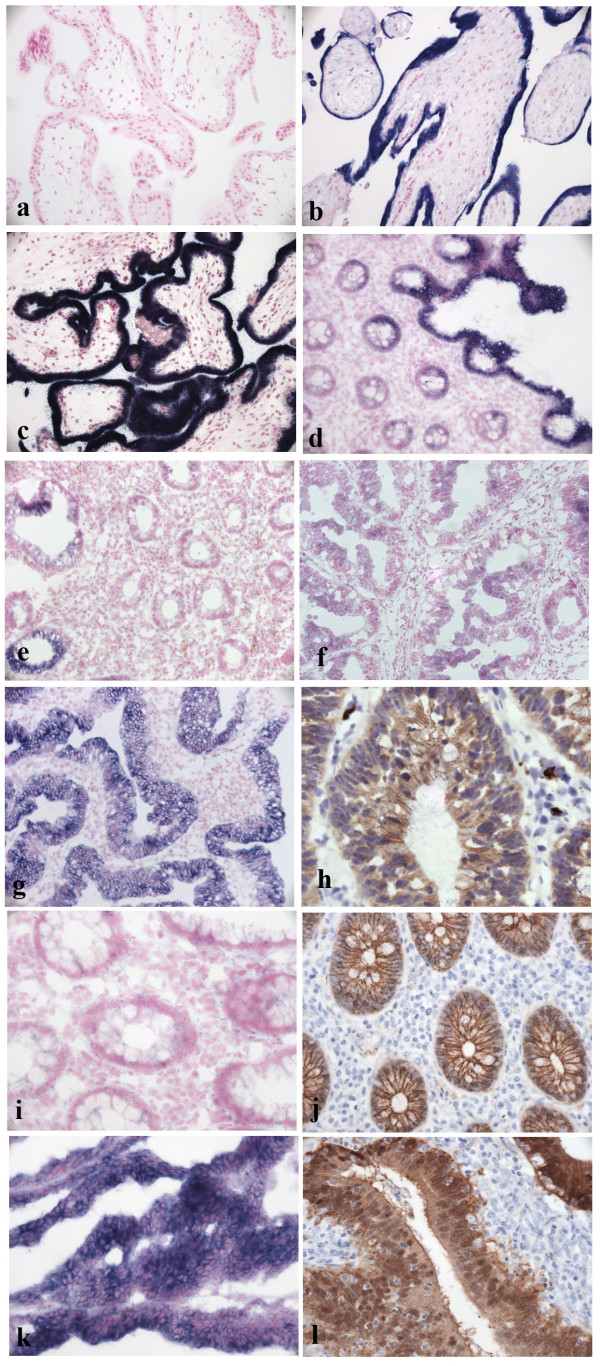
*RNA in situ hybridization and IHC analysis of colorectal cancer cases*. Sections from sporadic/familial colorectal cancer and placenta (as positive control) were hybridised with Dig-labelled PSG9 RNA probes. Both *PSG2 *(b) and *PSG9 *(c) were expressed at a high level in placental tissue. Sense-probes were used as a negative control on placental tissue (a). In microscopic normal epithelial cells from FAP cases, *PSG9 *expression was detected at the top of crypt (d) (see discussion). *PSG9 *transcripts (shown as dark blue) were detected at very low levels in normal mucosa (e), adenomas (f), while high expression was detected in tumour cells from the same FAP case (g). In contrast to sporadic cases, *PSG9 *was detected in normal appearing mucosa in some FAP cases with *APC *germline mutations, suggesting that dose and level of APC have an impact on PSG9 levels in cells (e, i). A high level of *PSG9 *was detected in a sporadic case (k), while corresponding normal tissue was negative (i). Tumours and corresponding normal tissue were also examined for β-catenin stabilization by immunostaining (h, j, l). As expected, high levels of β-catenin were detected in all sporadic colorectal tumours (l), while the protein level was less intense in FAP cases (h) where *PSG9 *up-regulation could be measured (g).

To determine the degree of PSG9 deregulation in colorectal cancer we employed RNA *in situ *hybridization. Digoxigenin (DIG)-labelled riboprobes were designed to recognize specifically PSG9 variant 1 (nt 776→1087) or all variants (nt 98→318) (Fig. 1a). Normal placental tissues were used as positive control where both *PSG9 *and another member of PSG family, *PSG2 *are highly expressed (Fig. 4b–c). Sense-probes were used as negative controls (Fig. 4a). PSG9 RNA transcripts were detected at higher levels and at earlier stages in FAP cases than sporadic colorectal cancer. Transcripts of *PSG9 *were detected in 78% (14/18) of adenomas from FAP cases with *APC *germline mutations (Fig. 4g). Of those, 50% also expressed *PSG9 *in the corresponding normal mucosa (Fig. 4d). However, the expression level was lower in the histologically normal tissue than in the corresponding adenomas and tumours (Fig. 4e–g). In all cases the expression level was much higher in tumours compared to corresponding normal mucosa or adenoma in FAP cases. The degree of *PSG9 *expression in the tumour samples appears to be related to differentiation, with highly differentiated cells expressing the highest levels. *PSG9 *expression was also detected in 70% (42/60) of sporadic colorectal cancers, while only 8% (5/60) of the corresponding normal mucosa showed even low levels of *PSG9 *expression (Fig. 4i, 4k). To examine whether *PSG9 *expression was specific for tumours, we examined expression of *PSG2 *in the same tumours which had exhibited deregulated *PSG9 *expression. *PSG2 *was expressed at very low levels in both tumour and normal mucosa from colorectal cancer cases. As indicated earlier, both PSG9 and PSG2 are highly expressed in placenta (Fig. 4b–c). In general, *PSG9 *could be detected at higher levels in FAP cases. Notably, *PSG9 *was expressed in the morphologically normal mucosa of FAP patients with APC germline mutations (Fig. 4d–e), while its expression was rarely detectable in normal mucosa of sporadic colorectal cancers using *in situ *hybridization (Fig. 4i). The expression pattern of *PSG9 *also varied between different tumours and cell lines although the significance of these differences is unclear (Fig. 2–4). Normal tissues, adenomas and tumours were also examined for nuclear β-catenin accumulation by immunostaining (Fig. 4h, j, l). Nuclear β-catenin could be detected in all sporadic tumours (Fig. 4l), while staining was less intense in the normal-appearing epithelium and adenomas in FAP cases where PSG9 was highly upregulated (Fig. 4g–h). In general, higher levels of β-catenin nuclear accumulation could be detected in sporadic cancer (Fig 4l) compared to FAP cases (Fig. 4h) while as expected no β-catenin stabilisation was observed in the corresponding normal tissues (Fig. 4j).

Next, we examined whether the *PSG9 *expressed in tumours was different from *PSG9 *expressed by placental cells during pregnancy. The coding sequences of all *PSG9 *variants of two colon cancer cell lines (SW620 and LoVo) and two sporadic colorectal cancer cases were cloned and sequenced. Two sequence variations were found in the non-coding region, while no changes were found in the coding region of PSG9. These results indicate that the PSG9 proteins expressed by placental cells and tumour cells have similar sequences and might have similar function, however, the level of expression of each PSG9 variant differed from those found in placenta.

### Effect of Wnt signal activation on PSG9 expression in the absence of APC mutation

**Figure 5:**
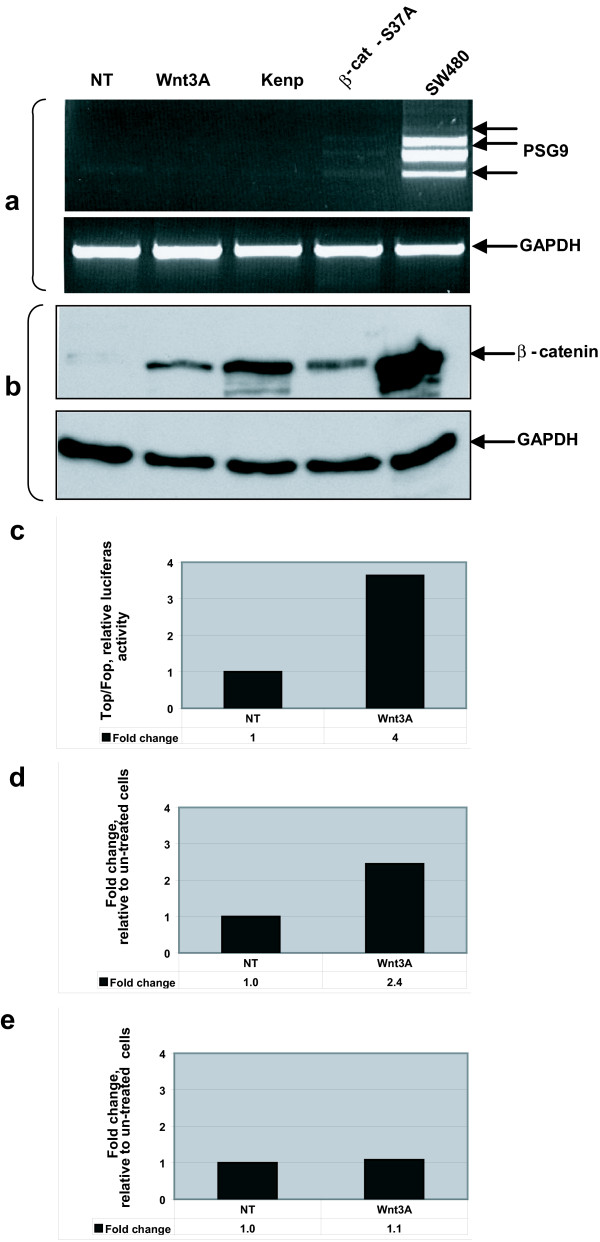
*PSG9 expression analysis in Wnt stimulated RKO cells*. To determine whether induction of Wnt signaling in cells with wild type APC could induce *PSG9 *expression, RKO cells with wild type APC and β-catenin were stimulated with either Wnt3a or Kenpaullone (Kenp). After 23 hrs treatment RNA and protein were extracted and processed for *PSG9 *transcripts expression level by semi-quantitative RT-PCR (a) and β-catenin accumulation by western blot analysis (b). Neither of the treatments nor the RKO-β-catenin^S37A ^(βcat-S37A) stable cell line which expressed constitutively active β-catenin in RKO cells caused expression of *PSG9 *(a). The SW480 colorectal cancer cell line was used as a positive control (a-b). The RKO cells responded to Wnt stimulation, since cells treated with Wnt3a showed 4-fold induction in luciferase activity compared to untreated cells (c). Axin2, a known downstream target of Wnt signaling, showed 2.4 fold up-regulation in expression as determined by quantitative PCR (d). No *PSG9 *transcript up-regulation was detected in these cells under these conditions (e). Each sample was analyzed in triplicate.

Nuclear localization of β-catenin has been shown to correlate with its transcriptional activity in cells. While SW480 cells with very high levels of β-catenin and *APC *mutations express high levels of *PSG9*, RKO cells which have previously been reported to express wild type APC and β-catenin [19,20] show no PSG9 expression (Fig. 2c and 5a–b). To investigate the potential role of β-catenin/Wnt3a up-regulation in the absence of APC mutations on PSG9 transcription, accumulation and nuclear localization of β-catenin in RKO cells was induced by treatment with recombinant Wnt3a, Kenpaullone, or using a stable RKO-β-catenin cell line which expresses constitutively a active mutant (βcat-S37A) form of β-catenin [21] (Fig. 5b). One day post-treatment, cells were prepared for luciferase assays and RNA extraction. Reporter gene assays were performed to examine the ability of these cells to respond to Wnt stimulation and quantitative PCR to investigate whether *Axin2*, a known Wnt signal downstream target gene was induced. Stimulation of the cells resulted in four-fold induction of luciferase activity (Fig. 5c), and a 2.4-fold increase in *Axin2 *transcript levels, indicating that these cells are able to respond to Wnt signaling and this signal induction can increase downstream target gene expression (Fig. 5d). We further examined the *PSG9 *expression level in these stimulated cells. No PSG9 induction in these cells could be detected (Fig. 5e). These results suggest that enhanced levels of β-catenin or Wnt signaling in cells, without concomitant defect in APC, is likely insufficient to induce PSG9 expression.

## Discussion

In this study, we have shown that PSG9 is ectopically expressed in colorectal cancer and this is most likely APC dependent, since abnormal expression can be detected as early as in normal appearing epithelial cells and adenomas of the FAP cases which carry *APC *germline mutations, while corresponding normal tissue in sporadic colorectal cancer tumors lack PSG9 expression. Given the increased expression of *PSG9 *in the mucosal cells of FAP patients that displayed lower β-catenin stabilisation compared to sporadic colorectal tumours in our study, it is possible that *PSG9 *is not directly regulated by the β-catenin/Wnt signalling pathway and that other molecules that regulate *PSG9 *expression are altered as a consequence of *APC *mutation. Notably, deregulation of PSG9 is detectable as early as in mucosa that appears histologically normal in FAP cases with *APC *germline mutations, suggesting that the dose and gradient of APC is important in PSG9 regulation. Functional analysis of APC protein has revealed a broad spectrum of activities for this molecule [22,23]. In normal-appearing epithelial cells in FAP, *PSG9 *was expressed mostly on the apical surface, while in tumours, expression was detected from the top to the base of crypts. Early expression of *PSG9 *even before adenoma formation at the top of the crypts in FAP cases, suggests that transformation process starts in cells at the top of the crypts which then gradually move downward (Fig. 4d). These observations are consistent with the "top-down" morphogenesis model of colorectal cancer [24].

*PSGs *exhibit sequence similarity to the carcinoembryonic antigen (CEA) family which, in turn, is a member of the immunoglobulin (Ig) superfamily. The *CEA *gene family can be divided into three subgroups; the *CEA *subgroup (12 genes), *PSG *subgroup (11 genes) and a pseudogene subgroup (6 genes) [25]. CEA is a widely used tumour marker, the main clinical utility of which is in monitoring clinical course of colorectal carcinoma after surgical resection [26]. Contrary to its name, CEA is expressed in normal adult tissue, as well as during fetal development. The role of CEA in normal human physiology is not well understood. Based on its structure, a number of functions have been suggested, including intracellular cell adhesion, signal transduction or signal transduction regulation. PSGs are secreted proteins and, in contrast to CEA, most PSGs are not expressed in normal colon epithelial cells. The main site for PSG production is the placental syncytiotrophoblasts during pregnancy [14]. Although PSGs were discovered more than three decades ago, their function is still unknown and the receptor(s) for these proteins has yet to be identified. Most PSGs have an RGD (Arg-Gly-Asp) motif in a conserved region in the N-terminal domain which suggest that these genes may function as adhesion recognition signals for integrins [27].

### Immune privilege of cancer cells

Several studies suggest that T-cells are capable of recognizing and responding to tumours in experimental conditions, yet most tumours are able to escape detection by the immune system. A common characteristic of cancer and the placenta is their ability to avoid immune reactivity. The capability of cancer cells to sidestep the body's immune reaction, is believed to be partly aided by the protective coating of the cells, which is largely composed of glycoproteins [28]. PSGs are heavily glycosylated proteins and the primary amino acid sequence is masked by the sugar modification which helps in avoid specific antibody recognition [29]. It has been suggested that PSG production by trophoblasts regulates maternal immune response to the fetus. It is also possible that PSG9 expressed by pre-malignant and cancer cells protect tumours from recognition by the body's immune defences. Another similarity between the respective milieu where both placenta and adenomas develop is their low-oxygen environment. Trophoblast invasion and placental development during the first trimester occurs in a low-oxygen environment, as the blood flow to the intervillous space is not yet established [30]. We found lower levels of *beta hemoglobin *(HBB) RNA in adenomas compared to normal mucosa in this study (Table 1) as well as in another gene profiling study we have performed (manuscript in preparation).

### Role of PSG9 in cancer

Most of the *PSG *subgroup (11 genes) are expressed. However, the existence of allelic variants with a stop codon in the N-domain of some PSGs, indicates that some individuals may not express all members of the PSG family [31]. The possible involvement of PSGs in cancer and their genetic variation may in part explain phenotypic divergence that exists in cancer cases with otherwise identical germline mutations. The RGD sequence motif in the N-terminal domain of most PSGs is also present in a variety of extracellular matrix proteins that bind to integrin receptors such as fibronectin and vitronectin [32]. It has been hypothesized that the PSGs (like most Ig superfamily members) are involved in adhesion/recognition processes. Another possibility is that upregulation of PSG9 might favour tumour development by causing a reversion of the monolayered adult colonic epithelium to an embryonic multilayered arrangement.

## Conclusion

Our results provide strong evidence that PSG9 deregulation in cells occurs early during adenoma-carcinoma formation. High levels and early deregulation of PSG9 in adenomas, as well as normal mucosa in some FAP cases, indicates the potent role of APC germline mutations that are often found in these cases. The precise role of PSG9 in carcinogenesis remains to be determined. However, early-onset over-expression of PSG9 in different types of cancer suggests that this gene may be considered as a valuable biochemical tumorigenesis marker. To elucidate whether the frequency of occurrence of elevated PSG9 could have clinical significance, further analysis of serum levels of PSG9 and also other PSGs are warranted. Since PSG9 is not found in the non-pregnant adult except in association with cancer, it may be useful as a biomarker for the early detection of cancers of various types.

## Abbreviations

CRC; colorectal cancer, FAP; familial adenomatous polyposis, APC; adenomatous polyposis coli, CEA; carcinoembryonic antigen, PSG; pregnancy specific glycoprotein, SAM; statistical analysis of microarrays.

## Competing interests

The author(s) declare that they have no competing interests.

## Authors' contributions

SS; designed and performed the study, and drafted the manuscript. JG; assisted with the microarray data analysis. RC; was involved in the analysis and interpretation of histopathology and reviewed the final manuscript. SG; coordinated data/material collection from clinic and reviewed the final manuscript. JRW; conceived the study, participated in design and coordination and helped draft the manuscript.

## Pre-publication history

The pre-publication history for this paper can be accessed here:



## Figures and Tables

**Table 1:** Genes upregulated in all adenomas compared to normal mucosa (p < 0.05).

**Name**	**Description**	**Gene ID**	**Fold change**
**PSG9**	pregnancy specific beta-1-glycoprotein 9	196828	2.59
**PPP2CB**	protein phosphatase 2 (formerly 2A), catalytic subunit, beta isoform	469697	1.71
**ITGA1**	integrin, alpha 1	212078	1.95
**NEDD8**	neural precursor cell expressed, developmentally down-regulated 8	220392	1.74
**ALAS2**	aminolevulinate, delta-, synthase 2 (sideroblastic/hypochromic anemia)	201112	1.98
**DCN**	decorin	197609	1.85
**JAG1**	jagged 1 (Alagille syndrome)	117734	2.21
**DPP6**	dipeptidylpeptidase 6	166550	1.86
**RNASE4**	ribonuclease, RNase A family, 4	201596	2.44
**XBP1**	X-box binding protein 1	213933	1.95
**CDC42**	cell division cycle 42 (GTP binding protein, 25 kDa)	214563	1.73
**MGC8407**	hypothetical protein MGC8407	179857	1.78
**NUP98**	nucleoporin 98 kDa	206345	2.02
**CDKN1A**	cyclin-dependent kinase inhibitor 1A (p21, Cip1)	470149	1.56
**IL1R2**	interleukin 1 receptor, type II	470402	1.54
**PIP5K1B**	phosphatidylinositol-4-phosphate 5-kinase, type I, beta	211877	1.64
**TAGLN2**	transgelin 2	152371	1.67
**TAF7**	TAF7 RNA polymerase II, TATA box binding protein (TBP)-associated factor	365930	1.81
**RAB25**	RAB25, member RAS oncogene family	149515	1.64
**ANAPC7**	anaphase-promoting complex subunit 7	487822	1.79
**MTCH2**	mitochondrial carrier homolog 2 (C. elegans)	138486	1.8
**HCA112**	hepatocellular carcinoma-associated antigen 112	207556	2.3
**BS69**	adenovirus 5 E1A binding protein	31337	1.69
**MGC29898**	hypothetical protein MGC29898	504671	1.65
**LOC124245**	hypothetical protein BC001584	194780	2.05
**FVT1**	follicular lymphoma variant translocation 1	115784	1.55
**FLJ20202**	FLJ20202 protein	142952	1.59
**ARHGDIA**	Rho GDP dissociation inhibitor (GDI) alpha	32555	1.63
**HEAB**	ATP/GTP-binding protein	212684	1.59
**VRK3**	vaccinia related kinase 3	488478	1.49
**HLA-DPA1**	major histocompatibility complex, class II, DP alpha 1	200735	1.48
**WFDC2**	WAP four-disulfide core domain 2	366323	1.85
**KIAA0241**	KIAA0241 protein	212216	1.83
**GPR161**	G protein-coupled receptor 161	489796	1.81
**SLAMF8**	SLAM family member 8	128371	1.99
**ARG99**	ARG99 protein	163160	1.7
**LOC170394**	hypothetical protein BC011630	154928	1.9

Differentially expressed genes identified in adenomas compared to corresponding normal tissue. In total, 84 genes showed significant differential expression in all three adenomas compared to normal-appearing epithelial cells (p < 0.05). Thirty-seven genes were up-regulated (Table 1), and 47 down-regulated (Table 2). PSG9 showed a consistent 2-fold over-expression in all adenomas compared to normal mucosa (p < 0.006).

**Table 2:** Genes downregulated in all adenomas compared to normal mucosa (p < 0.05).

**Name**	**Description**	**Gene ID**	**Fold change**
**AFP**	alpha-fetoprotein	428098	1.91
**GFAP**	glial fibrillary acidic protein	382693	2.23
**HBB**	hemoglobin, beta	148425	1.74
**CLU**	clusterin (complement lysis inhibitor, SP-40,40, sulfated glycoprotein 2	343293	1.75
**VDR**	vitamin D (1,25- dihydroxyvitamin D3) receptor	344295	2.09
**MGP**	matrix Gla protein	502239	1.97
**IGFBP2**	insulin-like growth factor binding protein 2, 36 kDa	471830	1.74
**PRKCE**	protein kinase C, epsilon	51986	3.39
**COX4I1**	cytochrome c oxidase subunit IV isoform 1	178644	1.89
**APOB**	apolipoprotein B (including Ag(x) antigen)	300017	1.97
**BIRC4**	baculoviral IAP repeat-containing 4	138505	2.15
**ITGAE**	integrin, alpha E (antigen CD103, human mucosal lymphocyte antigen 1; alpha polypeptide)	358848	2.3
**GAS1**	growth arrest-specific 1	341345	1.47
**GATA3**	GATA binding protein 3	148627	1.47
**VEGFB**	vascular endothelial growth factor B	167296	2.01
**ADIPOR2**	adiponectin receptor 2	24286	1.98
**RUFY2**	RUN and FYVE domain containing 2	161523	2
**LRP8**	low density lipoprotein receptor-related protein 8, apolipoprotein e receptor	134714	2.1
**SHMT2**	serine hydroxymethyltransferase 2 (mitochondrial)	51583	1.96
**TXK**	TXK tyrosine kinase	147839	1.7
**LOC51315**	hypothetical protein LOC51315	503300	1.39
**UGCGL2**	UDP-glucose ceramide glucosyltransferase-like 2	32716	3.02
**MGC5178**	hypothetical protein MGC5178	235090	1.73
**IPO9**	importin 9	233100	1.71
**KBRAS2**	I-kappa-B-interacting Ras-like protein 2	5091	2.05
**SLC35F2**	solute carrier family 35, member F2	42703	2.55
**FLJ33761**	hypothetical protein FLJ33761	23095	2.07
**ZNF198**	zinc finger protein 198	153735	1.75
**PAG**	phosphoprotein associated with glycosphingolipid-enriched microdomains	487926	2
**FLJ40432**	hypothetical protein FLJ40432	23334	2.12
**LOC114926**	hypothetical protein BC013035	270038	1.64
**LOC51277**	Ras-associated protein Rap1	44081	1.92
**FLJ20360**	hypothetical protein FLJ20360	270110	1.61
**LOC56931**	hypothetical protein from EUROIMAGE 1967720	145011	1.8
**LBP-32**	leader-binding protein 32	195784	1.81
**SATB2**	SATB family member 2	26583	1.78
**CTDSP1**	CTD (carboxy-terminal domain, RNA polymerase II, polypeptide A) small phosphatase 1	131828	2.13
**OSBPL2**	oxysterol binding protein-like 2	305402	1.81
**FLJ38991**	hypothetical protein FLJ38991	31673	2.16
**MGC12972**	hypothetical protein MGC12972	256553	1.69
**ZNF496**	zinc finger protein 496	32065	1.81
**FLJ35936**	hypothetical protein FLJ35936	33409	2
**C14orf141**	chromosome 14 open reading frame 141	161520	1.68
**SGPP2**	sphingosine-1-phosphate phosphotase 2	46316	1.94
**DKFZp313N0621**	hypothetical protein DKFZp313N0621	258616	2.22
**LOC285550**	hypothetical protein LOC285550	250675	1.89
**MGC52498**	hypothetical protein MGC52498	683068	1.85

**Table 3:** Genes differentially expressed within different adenomas compared to corresponding normal mucosa.

**Gene's name**	**Description**	**A1**	**A2**	**A3**
GAS1	growth arrest-specific 1	↓	↑	↓
IGFBP2	insulin-like growth factor binding protein 2	↓	↑	↓
MAPRE3	microtubule-associated protein, RP/EB family, member 3	↑	↓	↓
ZFYVE20	zinc finger, FYVE domain containing 20	↑	↑	↓
FLJ11848	hypothetical protein FLJ11848	↓	↓	↑
KIAA1750	KIAA1750 protein	↓	↓	↑
KIAA1941	KIAA1941 protein	↓	↑	↓

Differentially expressed genes identified in adenomas compared to corresponding normal tissue. In total, 84 genes showed significant differential expression in all three adenomas compared to normal-appearing epithelial cells (p < 0.05). Thirty-seven genes were up-regulated (Table 1), and 47 down-regulated (Table 2). PSG9 showed a consistent 2-fold over-expression in all adenomas compared to normal mucosa (p < 0.006).

## References

[B1] Nishisho I, Nakamura Y, Miyoshi Y, Miki Y, Ando H, Horii A, Koyama K, Utsunomiya J, Baba S, Hedge P (1991). Mutations of chromosome 5q21 genes in FAP and colorectal cancer patients. Science.

[B2] Gryfe R, Gallinger S (2001). Microsatellite instability, mismatch repair deficiency, and colorectal cancer. Surgery.

[B3] Kinzler KW, Nilbert MC, Vogelstein B, Bryan TM, Levy DB, Smith KJ, Preisinger AC, Hamilton SR, Hedge P, Markham A (1991). Identification of a gene located at chromosome 5q21 that is mutated in colorectal cancers. Science.

[B4] Kinzler KW, Vogelstein B (1996). Lessons from hereditary colorectal cancer. Cell.

[B5] Korinek V, Barker N, Morin PJ, van Wichen D, de Weger R, Kinzler KW, Vogelstein B, Clevers H (1997). Constitutive transcriptional activation by a beta-catenin-Tcf complex in APC-/- colon carcinoma. Science.

[B6] Woodgett JR (2001). Judging a protein by more than its name: GSK-3. Sci STKE.

[B7] Ausubel MF, Brent R, Kingston RE, Moore DD, Seidman JG, Smith JA, Struhl K (1994). Current Protocoles in Molecular Biology.

[B8] Cohen P, Goedert M (2004). GSK3 inhibitors: development and therapeutic potential. Nat Rev Drug Discov.

[B9] Veeman MT, Slusarski DC, Kaykas A, Louie SH, Moon RT (2003). Zebrafish prickle, a modulator of noncanonical Wnt/Fz signaling, regulates gastrulation movements. Curr Biol.

[B10] Plouzek CA, Watanabe S, Chou JY (1991). Cloning and expression of a new pregnancy-specific beta 1-glycoprotein member. Biochemical & Biophysical Research Communications.

[B11] Tatarinov YS, Hobes CA (1970). Immunological identification of a new b1-globulin in the blood serum of pregnant women. Byull EKsp Biol Med.

[B12] Bohn H (1971). Detection and characterization of pregnancy proteins in the human placenta and their quantitative immunochemical determination in sera from pregnant women. Arch Gynakol.

[B13] Lin TM, Halbert SP, Spellacy WN (1974). Measurement of pregnancy-associated plasma proteins during human gestation. J Clin Invest.

[B14] Zhou GQ, Baranow V, Zimmermann W, Grunert F, Erhard B, Mincheva-Nilsson L, Hammarström S, Tompson J (1997). Highly specific monoclonal antibody demonstrates that pregnancy-specific glycoprotein (PSG) is limited to syncytiotrophoblast in human early and term placenta.. Placenta.

[B15] Zimmermann W, Webe B, Ortlieb B, Rudert F, Schempp W, Fiebig HH, Shively JE, Von Kleist S, Thompson JA (1988). Chromosomal localization of the carcinoembryonic antigen gene family and differential expression in various tumors.. Cancer Research.

[B16] Borjigin J, Tease LA, Barnes W, Chan WY (1990). Expression of the pregnancy-specific beta 1-glycoprotein genes in human testis. Biochemical & Biophysical Research Communications.

[B17] Horne CH, Towle CM, Milne GD (1977). Detection of pregnancy specific beta1-glycoprotein in formalin-fixed tissues.. J Clin Pathol.

[B18] Shupert WL, Chan WY (1993). Pregnancy specific beta 1-glycoprotein in human intestine. Molecular & Cellular Biochemistry.

[B19] Da Costa LT, He TC, Yu J, Sparks AB, Morin PJ, Polyak K, Laken S, Vogelstein B, Kinzler KW (1999). CDX2 is mutated in a colorectal cancer with normal APC/beta-catenin signaling. Oncogene.

[B20] Sparks AB, Morin PJ, Vogelstein B, Kinzler KW (1998). Mutational analysis of the APC/beta-catenin/Tcf pathway in colorectal cancer. Cancer Res.

[B21] Deng J, Miller SA, Wang HY, Xia W, Wen Y, Zhou BP, Li Y, Lin SY, Hung MC (2002). beta-catenin interacts with and inhibits NF-kappa B in human colon and breast cancer. Cancer Cell.

[B22] Fearnhead NS, Britton MP, Bodmer WF (2001). The ABC of APC. Human Molecular Genetics.

[B23] Näthke IS (2004). The adenomatous polyposis coli protein: the Achilles heel of the gut epithelium. Annu Rev Cell Dev Biol.

[B24] Shih IM, Wang TL, Traverso G, Romans K, Hamilton SR, Ben-Sasson S, Kinzle KW, Vogelstein B (2001). Top-down morphogenesis of colorectal tumors.. Proc Natl Acad Sci.

[B25] Olsen A, Teglund S, Nelson D, Gordon L, Copeland A, Gorgescu A, Garrano A, Hammarström S (1994). Gene organization of the pregnancy-specific glycoprotein region on human chromosome 19: Assembly and analysis of a 700 kb cosmid conting sapnning the region.. Genomic.

[B26] Thomson DM, Krupey J, Freedman SO, Gold P (1969). The radio immunoassay of circulating carcinoembryonic antigen of the human digestive system.. Proc Natl Acad Sci USA.

[B27] Hammarström S (1999). The carcinoembryonic antigen (CEA) family: structures, suggested functions and expression in normal and malignant tissues. Seminars in Cancer Biology.

[B28] Currie GA, Bagshawe KD (1967). The masking of antigens on trophoblast and cancer cells. Lancet.

[B29] Chen H, Plouzek CA, Liu JL, Chen CL, Chou JY (1992). Characterization of a major member of the rat pregnancy-specific glycoprotein family. DNA Cell Biol.

[B30] Rodesch F, Simon P, Donner C, Jauniaux E (1992). Oxygen measurements in endometrial and trophoblastic tissues during early pregnancy. Obstet Gynecol.

[B31] Teglund S, Olsen A, Khan WN, Frangsmyr L, Hammarström S (1994). The pregnancy-specific glycoprotein (PSG) gene cluster on human chromosome 19: fine structure of the 11 PSG genes and identification of 6 new genes forming a third subgroup within the carcinoembryonic antigen (CEA) family.. Genomics.

[B32] Mansfield BC, Rutherfurd KJ, Chou JY (1995). A motif in PSG11s mediates binding to a receptor on the surface of the promonocyte cell line THP-1. Mol Endocrinol.

